# Efficacy of transumbilical laparoendoscopic single-site surgery versus multi-port laparoscopic surgery for endometrial cancer: a retrospective comparison study

**DOI:** 10.3389/fonc.2023.1181235

**Published:** 2023-08-28

**Authors:** Xiaolin You, Yanyun Wang, Ying Zheng, Fan Yang, Qiao Wang, Ling Min, Kana Wang, Na Wang

**Affiliations:** ^1^ Department of Obstetrics and Gynaecology, West China Second University Hospital, Sichuan University, Chengdu, Sichuan, China; ^2^ Key Laboratory of Birth Defects and Related Diseases of Women and Children (Sichuan University), Ministry of Education, West China Second University Hospital, Sichuan University, Chengdu, Sichuan, China; ^3^ Laboratory of Molecular Translational Medicine, Center for Translational Medicine, West China Second University Hospital, Sichuan University, Chengdu, Sichuan, China

**Keywords:** transumbilical laparoendoscopic single-site surgery, multi-port laparoscopic surgery, minimally invasive surgery, sing-port laparoscopy, endometrial cancer, sentinel lymph node biopsy

## Abstract

**Background:**

Although single-port laparoscopy surgery has been evaluated for several years, it has not been widely adopted by gynecologic oncologists. The objective was to compare the perioperative outcomes and survival of endometrial cancer (EC) patients undergoing transumbilical laparoendoscopic single-site surgery (TU-LESS) with multi-port laparoscopic surgery (MLS).

**Materials and methods:**

This is a retrospective comparative monocentric study including patients treated between December 2017 and October 2021. The perioperative outcomes and survival of EC patients who had surgery via TU-LESS or MLS were compared, by propensity matching.

**Results:**

A total of 156 patients were included (TU-LESS vs. MLS: 78 vs. 78). The conversion rate of TU-LESS and MLS was 5.13% and 2.56%, respectively (*P*=0.681). The operation time was comparable between the two groups [207.5min (180-251) vs. 197.5min (168.8-225), *P*=0.095]. There was no significant difference between the two groups in exhaustion time, perioperative complications, or postoperative complications. While, the TU-LESS group had a shorter out-of-bed activity time [36 hours (24-48) vs. 48 hours (48-72), *P*<0.001] and a lower visual analog pain scale 36 hours after surgery [1 (1-2) vs. 2 (1-2), *P*<0.001] than the MLS group. The length of hospital stay was similar in the two groups [5(4-6) vs. 5(4-5), *P*=0.599]. Following surgery, 38.5% of the TU-LESS patients and 41% of the MLS patients got adjuvant therapy (*P*=0.744). The median follow-up time for TU-LESS and MLS cohorts was 45 months (range: 20-66) and 43 months (range: 18-66), respectively. One TU-LESS patient and one MLS patient died following recurrence. The 4-year overall survival was similar in both groups (98.3% vs. 98.5%, *P*=0.875).

**Conclusion:**

TU-LESS is a feasible and safe option with comparable perioperative outcomes and survival of MLS in endometrial cancer. With the growing acceptance of sentinel lymph node biopsy, TU-LESS of endometrial cancer may be a viable option for patients and surgeons.

## 1 Introduction

Endometrial cancer (EC) is the second most common malignant tumor of the female reproductive system in China, with an incidence rate of 10.28/100000 and a mortality rate of 1.9/100000 (1, 2). The pathogenesis of EC is related to continuous estrogen exposure, carrying genetic susceptibility genes, old age, metabolic abnormalities, obesity, diabetes, and so on (1–3). Surgery is the primary modality used for both staging and treatment for endometrial cancer patients. Standard surgical treatment for endometrial cancers includes total hysterectomy (TLH) with bilateral salpingo-oophorectomy (BSO) with or without lymphadenectomy for newly diagnosed endometrial cancers (4–6).

Minimally invasive surgery is currently the preferred surgical path for EC patients, and number of studies had shown feasibility of minimally invasive surgery in lymphadenectomy of gynecologic cancers. Since the adoption of single-port laparoscopy (SPL) in gynecologic oncology was first described at Cleveland Clinic Foundation in 2009 (7), the role and potential benefits of SPL in EC surgery have been described in the literature. SPL has been shown as safe and effective as traditional laparoscopy in gynecologic surgery, with lower operative morbidity, decreased post-operative pain, a shorter recovery period, and superior cosmesis (8–15).

So far, the largest number of patients undergoing single-port laparoscopy by a surgeon included 110 consecutive endometrial cancer patients undergoing full staging with bilateral pelvic and para-aortic lymphadenectomy (11). It reported that single-port laparoscopic staging of endometrial cancer is a safe and feasible technique to introduce into gynecologic oncology practice. However, this study did not include contemporaneous cases, or historical cases undergoing multi-port laparoscopy. Thus, there is still a need to further understand the impact of single-port laparoscopy on uterine cancer when hysterectomy and, or pelvic and para-aortic lymphadenectomy is consecutively utilized in all cases.

The objective of this study is to summarize our accumulative 4 years of consecutive transumbilical laparoendoscopic single-site surgery (TU-LESS) of endometrial cancer that included hysterectomy with bilateral salpingo-oophorectomy, as well as performing/or not pelvic lymphadenectomy or para-aortic lymphadenectomy by one experienced gynecologist and to compare the perioperative outcomes and survival with concurrent multi-port laparoscopic surgery (MLS) conducted by the other senior surgeons with extensive expertise in laparoscopic surgery for gynecological malignant tumors in our hospital.

## 2 Materials and methods

### 2.1 Experimental design

We conducted a retrospective analysis from a prospectively maintained database of endometrial cancer in our institute between December 2017 and October 2021. The goal of this study is to compare the perioperative outcomes, postoperative complications, and oncological outcomes in endometrial cancer between TU-LESS and MLS. Ethical approval was obtained from the Institutional Ethics Committee of our Hospital. Inclusive criteria included: (1) patients diagnosed as EC by pathology before or during operation; (2) the operation method was TLH + BSO ± pelvic/paraaortic lymph node dissection (PLND/PALND). The following conditions were used as an exclusion criterion: (1) metastatic endometrial carcinoma or EC in combination with other malignant tumors; (2) insufficient clinicopathological data; (3) patients who were lost to follow-up. Endometrial cancer patients who had contemporaneous multi-port laparoscopic surgery were included by propensity matching with TU-LESS, including the matching types of surgery (TLH+BSO, TLH+BSO+PLND, or TLH+BSO+PLND+PALND), body mass index BMI ± 4 kg/m^2^, and history of abdominal surgery (proportion ± 20% scale). Finally, a total of 156 patients were included (TU-LESS vs. MLS: 78 vs. 78).

The same surgeon performed all the TU-LESS surgeries, a senior gynecologist with extensive experience in laparoscopic surgery for gynecological malignant tumors, and expertise in single-site laparoscopic operations. All of the MLS chief surgeons were senior surgeons with extensive expertise in laparoscopic surgery for gynecological malignant tumors in our institute. A telephone follow-up was used to gauge incision satisfaction, and a score of 1~10 was assigned (0, absolutely dissatisfied; 10, completely satisfied). The primary endpoints were peri-operative outcomes, and the secondary endpoint was the survival of patients. Disease outcomes were collected using the last institutional follow-up, recurrence, and death. Data was collected for demographics, pathologic information, adjuvant treatment, and disease status. The operative duration was defined as the time from skin incision start to closure. BMI (kg/m^2^) was categorized by World Health Organization criteria. Intraoperative complications were identified as injuries to the bowel, bladder, ureter, nerves, or vascular systems. Postoperative complications were classified as fever, urine retention, urinary tract infection, venous thromboembolism, delayed bowel, ureteral, or bladder injury, incisional cellulitis, deep wound infection, vaginal cuff dehiscence, or readmission within 30 days following surgery. Any hernia discovered clinically during postoperative surveillance was referred to as an incisional hernia. Recurrence was defined as the local, regional, or distant re-emergence of the illness that was detected by histological sample or imaging. Disease-free survival (DFS) was defined as the time from surgery until the time of first recurrence, and patients without recurrence were censored at the time of the last follow-up or time of non–disease-related death. The time from operation until death was referred to as overall survival (OS). Data on live patients were censored at the last follow-up visit.

### 2.2 Surgical procedures

A commercially available 4-channel, the single-port device was used during TU-LESS surgery (Kangji, Hangzhou). As previously stated (16, 17), the system comprises two 5-mm cannulas, one 10-mm cannula, and one 12-mm cannula. Following general anesthesia, each patient was put in a supine position and disinfected. A simple uterine manipulator was placed by an assistant through the vagina to aid expose the surgical field of vision. The umbilicus was sliced lengthwise for about 2cm in the center. The multichannel port system was used to create a pneumoperitoneum with a CO2 pressure of 12mm hg (1mm hg=0.133kpa). The port cannulas were used to implant the laparoscopic lens and surgical equipment.

First, all patients underwent cytologic washing of the pelvic and peritoneal cavities. After an overall exploratory analysis of the pelvic and abdominal viscera, the patient’s position was changed to the Trendelenburg position. The initial parts of bilateral fallopian tubes were coagulated at the beginning of the operation. All patients underwent TLH, BSO, ± PLND/PALND. Those with difficulty in intraoperative exposure were exposed to “Zheng’s 4C Suspension” to expose the pelvic and the para-aortic lymph nodes (17) and to avoid bladder injury. Finally, the vaginal cuff was closed by absorbable barbed suture, a T-shaped drainage tube through the vagina could be retained based on the intraoperative situation, and the umbilical incision was closed layer by layer using “Zheng’s anchor suturing technique” (17, 18).

The multi-port cohort used 4-5 puncture holes to complete the surgery, including a 10mm puncture hole in the umbilicus or 3cm above for the laparoscopic lens insertion, and the other 3-4 5mm cannulas were placed in the lower abdomen, for the surgical instruments’ placement. The operation steps were the same as that of TU-LESS. An assistant helped to expose the operation field, and the abdominal wall puncture holes were closed with absorbable sutures after the operation. The surgical energy instruments used in the two groups were a unipolar electric hook and an ultrasonic dissector. All patients were anesthetized with endotracheal intubation, and the average pressure in the abdominal cavity was 12mmhg (1mmhg = 0.133kpa).

### 2.3 Postoperative management

After surgery, 96% of patients in the TU-LESS cohort and 98% of patients in the MLS chose to use patient-controlled analgesia for 48 hours. The patient-controlled analgesia regimen consisted of tramadol 1200mg in normal saline (total volume, 204ml) and was programmed to deliver 2ml/h as a basal infusion rate and 1ml/demand with a 60-minute lockout, or butorphanol tartrate 10mg, sufentanil citrate 100ug in normal saline (total volume, 200ml) and was programmed to deliver 2ml/h as a basal infusion rate and 2ml/demand with a 20-minute lockout. Postoperative pain was recorded using the visual analog pain scale (VAS), with 0 representing no pain and 10 representing extreme pain every 12 hours, for 36 hours after surgery. If the pain score was greater than 3 or the patient requested, additional analgesics were provided.

Regular diet was restored on the first day after the operation. The retention time of the urinary catheter was related to the extent of the hysterectomy. The drainage tube would be removed if the daily drainage flow was less than 30ml. Recommendation for discharge was made following spontaneous urination, re-establishment of the regular diet, exhaustion, and no symptoms of fever and infection. Adjuvant treatment was recommended for high-risk patients according to the postoperative pathological result. After the completion of primary treatment, clinical follow-up was scheduled at 3-month intervals for two years, then at 6-month intervals for the subsequent three years, then annually thereafter.

### 2.4 Statistical analysis

Demographic characteristics (age, body mass index, comorbidities such as hypertension, diabetes, history of abdominal surgery), surgical outcomes (operating time, scopes, and numbers of lymph node dissection), histological type, FIGO stage and follow-up results (early and late complications, recurrence or death) were recorded and compared between the 2 groups.

All data were statistically analyzed by SPSS 23 software. Continuous measures meeting normal distributions were expressed as mean ± standard deviation and were compared between the groups using an independent T-test. Data that didn’t meet normal distributions were expressed as median (ranks) and were compared between the groups using Mann-Whitney U tests. Categorical factors were summarized using frequencies and percentages and compared by Chi-square test or Fisher exact test. Kaplan-Meier method were used to compare the survival of patients. The difference was statistically significant with a *P*-value of less than 0.05.

## 3 Results

### 3.1 Sample characteristics

Between December 2017 and October 2021, 82 patients with endometrial cancer received TU-LESS surgery by the same surgeon on our team. Four of them were excluded (1 patient with a preoperative diagnosis of adenocarcinoma of the cervix and postoperative confirmation of endometrioid adenocarcinoma of the lower uterine body, who subsequently underwent a complementary double oophorectomy; 2 patients transferred to our institution with incomplete clinicopathological data; and 1 patient who was lost to follow-up after surgery). Finally, 78 EC patients treated with TU-LESS were included in the study. An additional 78 patients with endometrial cancer who underwent multiport laparoscopic surgery at the same time were enrolled, by propensity matching. **Table 1** shows the clinical and pathological characteristics of the 156 subjects. The TU-LESS group’s mean age and BMI were 50.3 ± 10.5 years and 24.4 ± 3.6 kg/m^2^, respectively, identical to the MLS group’s age of 52.6 ± 7.8 years and BMI of 24.2 ± 3.0 kg/m^2^. There was no difference in age, BMI, ASA classifications, history of abdominal surgery, menopause, medical comorbidities, FIGO stages, histologic subtypes, or tumor grades between the two groups (**Table 1**).

**Table 1:** Clinical and pathological characteristics of the 156 patients.

		TU-LESS(n=78)	MLS(n=78)	*P* value
Age, y		50.3±10.5	52.6±7.8	0.124
BMI, kg/m2		24.4±3.6	24.2±3.0	0.78
ASA				0.415
	I/II	72(92.3%)	69(88.5%)	
	III	6(7.69%)	9 (11.5%)	
History of abdominal surgery		41	41	1
Menopause		34(43.6%)	43(55.1%)	0.15
Medical comorbidities				
	Diabetes mellitus, DM	6(7.69%)	6(7.69%)	
	Hypertension, HTN	13(16.7%)	21(16.7%)	
	DM & HTN	3(3.85%)	3(3.85%)	
FIGO stage				0.608
	I	63(80.8%)	61(78.2%)	
	II	2(2.56%)	2(2.56%)	
	III	12(15.4%)	10(12.8%)	
	IV	1(1.28%)	2(2.56%)	
Histology				0.657
	Endometrioid	67(85.9%)	65(83.3%)	
	Others	11(14.1%)	13(16.7%)	
Grading				0.901
	G1	65(83.3%)	66(84.6%)	
	G2	3(3.85%)	2(2.56%)	
	G3	10(12.8%)	10(12.8%)	
LVSI		12 (15.4%)	8(10.3%)	0.338
Cytology		3(3.85%)	2(2.56%)	0.901

ASA, American Society of Anesthesiologists; LVSI, lymphovascular space invasion; FIGO, International Federation of Gynecology and Obstetrics.

### 3.2 Perioperative outcomes of the two groups

The comparison of perioperative outcomes of the two groups is shown in **Table 2**. The extent of the hysterectomy and the number of lymph nodes removed in the two groups were not significantly different. Four patients (5.13%) in the TU-LESS group were converted to MLS surgery/laparotomy because of intraoperative injury to the left external iliac vein, inferior vena cava, abdominal aorta, or severe pelvic adhesion. Two cases (2.56%) in the MLS group were converted to open surgery, one due to small intestine damage and the other due to inferior mesenteric artery injury.

**Table 2:** Perioperative and postoperative outcomes between the two groups.

	TU-LESS(n=78)	MLS(n=78)	*P* value
Extent of hysterectomy			0.574
Extrafascial	73 (%)	73 (%)	
Modified-radical	4 (%)	5 (%)	
Radical	1(%)	0(%)	
Scopes of lymph node resection			1
Hysterectomy/BSO ±variables^a^	3(3.85%)	3(3.85%)	
Hysterectomy/BSO/PLND±variables^a^	47(60.3%)	47(60.3%)	
Hysterectomy/BSO/PLND+PALND ± variables^a^	28(35.9%)	28(35.9%)	
Operative time, min	207.5(180-251)	197.5 (168.8-225)	0.095
Estimated blood loss, ml	100 (50-100)	100 (50-200)	0.324
Conversion to LPS/LPT, n (%)	4(5.13%)	2(2.56%)	0.681
intraoperative complications, n (%)	3(3.85%)	4(5.13%)	1
Drainage tube removal time, d	3 (2-5)	3 (3-4)	0.463
Indwelling catheter time, d	3 (2-4)	4 (3-4)	<0.001
Exhaust time, h	36(36-60)	38(36-60)	0.611
Time of activity, h	36(24-48)	48(48-72)	<0.001
Length of hospital stay, d	5 (4-6)	5 (4-5)	0.599
Lymph nodes retrieved	26(19-35)	29(20-39)	0.194
No. of pelvic nodes removed	23(16-28)	25(19-31)	
No. of aortic nodes removed	2(0-6)	2(0-7)	
Postoperative VAS pain score			
12h after surgery	2(1-2)	2(1-3)	0.21
24h after surgery	2(1-2)	2(1-2)	0.363
36h after surgery	1(1-2)	2(1-2)	0.001
Reoperation within 48h	0	0	
Readmission within 30d	0	1(%)	1
postoperative complications within 30d, n (%)	7(8.97%)	8(10.26%)	0.786
Incisional hernia	0	0	
Incision satisfaction score	10(9,10)	9(9,10)	<0.001

VAS, visual analog pain scale; ^a^Omental and/or peritoneal biopsy, appendectomy in case of special histological type, sentinel lymph node biopsy.

There was no difference in median operation duration [LESS 207.5min (180-251) vs. MLS 197.5min (168.8-225), *P*=0.095], estimated blood loss (100ml (50–100) vs. 100ml [50-200], *P*=0.324), or lymph node yield [26 (19-35) vs. 29(20-39), *P*=0.194] between the TU-LESS and the MLS group. The TU-LESS group had substantially shorter catheter indwelling duration [3 days (2-3) vs. 4 days (3-4), *P*<0.001] and out-of-bed activity time [38 hours (24-48) vs. 48 hours (48-72), *P*<0.001]. And there was no significant difference in intraoperative complications, exhaustion time, postoperative complications, or drainage tube removal time between the two cohorts. Visual analog pain scale (VAS 0~10 score, 0 was no pain and 10 was agonizing pain) was evaluated at 12 hours, 24 hours, and 36 hours after surgery. VAS at 36 hours of the TU-LESS group was lower than that of the MLS group [1 (1-2) vs. 2 (1-2), *P*<0.001]. And the length of hospital stay was similar in the two cohorts [5 (4-6) vs. 5 (4-5), *P*=0.599] (**Table 2**).

### 3.3 Perioperative outcomes of TU-LESS and MLS in different surgical types

**Table 3** compares the patient characteristics and perioperative parameters across the two groups by different surgery types (TLH+BSO, TLH+BSO+PLND, and TLH+BSO+PLND+PALND). There were 3, 47, and 28 patients in the three types, respectively. The BMI, history of abdominal surgery, expected blood loss, intraoperative and postoperative complications, and surgical conversion did not vary statistically in the two groups. And the operation time of the three types of surgery was comparable between the two cohorts [LESS vs. MLS, TLH+BSO: 140 (140-160) vs. 130 (95-145), P=0.4; TLH+BSO+PLND: 190 (175-220) vs. 180 (160-210), *P*=0.068; TLH+BSO+PLND+PALND: 264.5 (215-330) vs. 242.5 (210-260), *P*=0.085] (**Table 3**).

**Table 3:** Perioperative outcomes between the two groups according to different types of surgery.

		Hysterectomy/BSO ±variables^a^	Hysterectomy/BSO/ PLND±variables^a^	Hysterectomy/BSO/ PLND+PALND ± variables^a^
Cases, n	LESS	3	47	28
	MLS	3	47	28
BMI	LESS	22.42±2.17	24.66±3.74	24.15±3.57
	MLS	24.17±3.95	24.36±3.08	24.05±3.08
Previous abdominal surgeries, n	LESS	1(33.3%)	26(55.3%)	14(50%)
	MLS	1(33.3%)	26(55.3%)	14(50%)
	*P* value	1	1	1
EBL, mL	LESS	50(50-50)	50(50-100)	100(100-200)
	MLS	50(20-200)	100(50-100)	100(62.5-200)
	*P* value	1	0.27	0.859
Operative time, min	LESS	140(140-160)	190(175-220)	264.5(215-330)
	MLS	130(95-145)	180(160-210)	242.5(210-260)
	*P* value	0.4	0.068	0.085
IO complications, n (%)	LESS	0	1(2.13%)	2(7.14%)
	MLS	0	1(2.13%)	3(10.7%)
	*P* value		1	1
Conversion to LPS/LPT, %	LESS	0	2(4.26%)	2(7,14%)
	MLS	0	1(2.13%)	1(3.57%)
	*P* value		1	1
Time to discharge, d	LESS	3(3-3)	4(4-5)	6(5-6)
	MLS	3(3-4)	5(4-5)	5(4-6)
	*P* value	0.7	0.041	0.074
PO complications n (%)	LESS	0	4(8.51%)	3(10.7%)
	MLS	0	5(10.64%)	3(10.7%)
	*P* value		1	1

^a^Omental and/or peritoneal biopsy, appendectomy in case of special histological type, sentinel lymph node biopsy.

### 3.4 Adjuvant treatments and follow-up

The adjuvant therapy and follow-up results of the two groups are described in **Table 4**. About 61.5% of TU-LESS patients did not receive postoperative adjuvant treatment, compared to 59% in the MLS group, with no significant difference between the two groups (*P*=0.744). Adjuvant chemotherapy was administered to 6.41% (n=5) of patients in the TU-LESS group vs. 10.3% (n=8) in the MLS cohort, with carboplatin and paclitaxel being the most often utilized medications. Concurrent chemoradiotherapy was given to nearly one-fifth of the TU-LESS patients (21.8%, n=17) and 26.9% of the MLS patients (n=21). Meanwhile, 8 TU-LESS patients and 3 MLS patients got radiation after surgery (vaginal brachytherapy, VBT; pelvic external beam radiotherapy, EBRT; or EBRT+VBT) (**Table 4**).

**Table 4:** Postoperative adjuvant treatments and survival data.

	TU-LESS (n=78)	MLS (n=78)	*P* value
Follow-up time (month)	41 (range, 16-62)	39 (range, 14-62)	0.886
Adjuvant therapy after surgery	30 (38.5%)	32 (41%)	0.744
Radiation	8	3	
Chemotherapy	5	8	
Concurrent chemoradiotherapy	17	21	
Recurrence	5 (6.41%)	3 (3.85%)	
4-year DFS (%)	94.7 ± 5.1	96.0 ± 4.5	0.488
Death	1 (1.28%)	1 (1.28%)	
4-year overall survival (%)	98.3 ± 3.3	98.5 ± 2.9	0.875

DFS, disease free survival; OS, overall survival; EBRT, external beam radiotherapy; VBT, vaginal beam radiotherapy.

**Figure 1:**
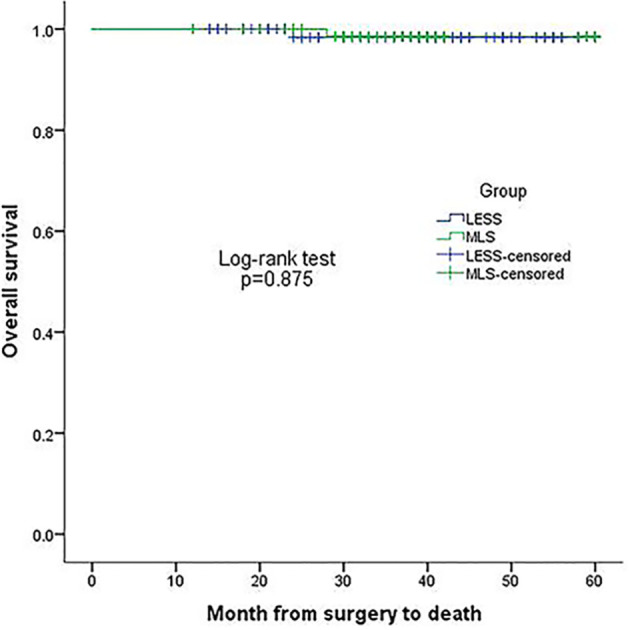
Kaplan-Meier plot of 4-year overall survival in the two groups.

**Figure 2:**
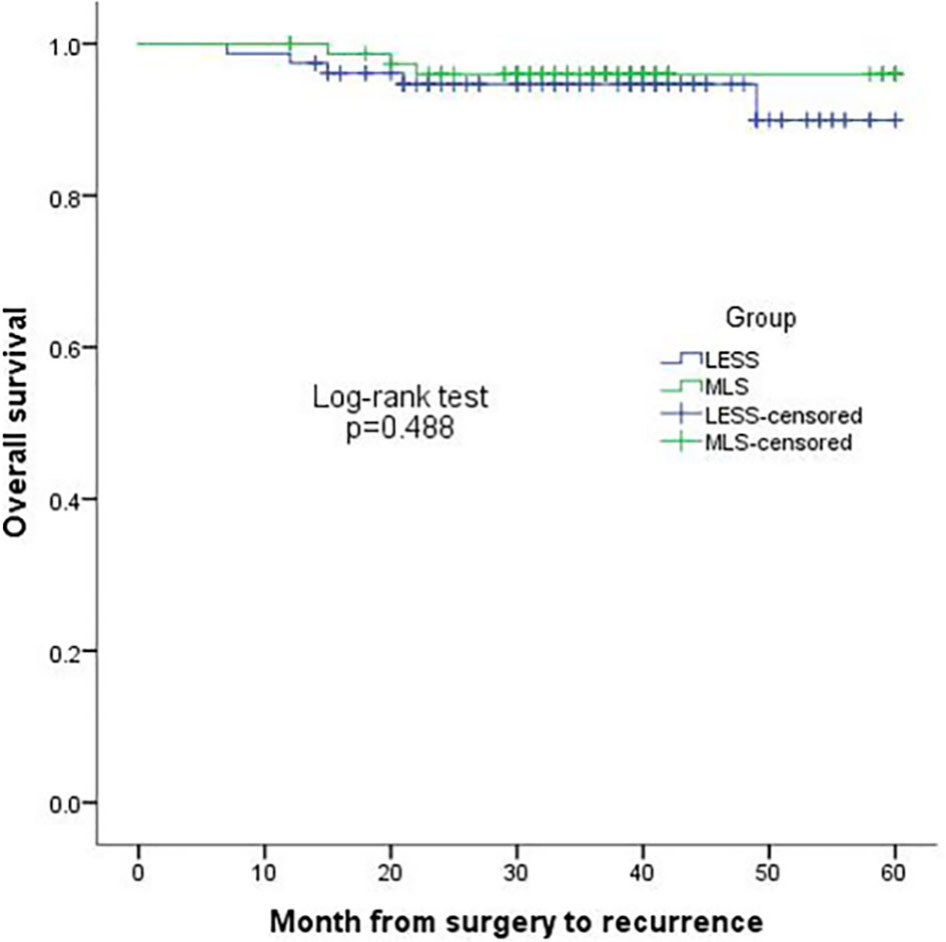
Kaplan-Meier plot of 4-year disease-free survival in the two groups.

The median follow-up period for the TU-LESS and MLS groups was 45months (range: 20-66) and 43 months (range: 18-66), respectively. In the TU-LESS group, five patients (6.41%) recurred, with one of them dying after recurrence; and in the MLS cohort, three patients (3.85%) recurred, with one of them passing away. The four-year DFS (TU-LESS vs. MLS: 94.7% vs. 96.0%, *P*=0.488) and four-year OS (98.3% vs. 98.5%, *P*=0.875) of the two cohorts were identical (**Figures 1**, **2**).

## 4 Discussion

Our data indicated the feasible and safe completion of EC surgery via transumbilical single-site laparoscopy in our consecutive case-matched control series. In this study, all TU-LESS operations were performed by a gynecological oncologist with extensive competence in multi-port laparoscopy and single-site laparoscopy. In the matched MLS group, the chief surgeons were senior gynecologists with extensive expertise in gynecological tumor endoscopic surgery. Patients who underwent TU-LESS had comparable perioperative results and oncological outcomes to those of MLS, while it is associated with less pain and a shorter stay in bed. The conversion rates in the TU-LESS and MLS groups were 5.13% and 2.56%, respectively, which were comparable with earlier studies (19).

The results of laparoendoscopic single-site surgery for endometrial cancer are similar to those of multi-port laparoscopic surgery, which is consistent with earlier reports in the literature. There were no differences between our two cohorts in terms of operative time, operative blood loss, postoperative hospitalization, or number of lymph nodes surgically resected (19–23). The rates of intraoperative and postoperative complications for EC patients undergoing MLS were reported to be 2.1~8.4% and 1.3~19.8% (20), whereas the rates of TU-LESS were 0~4% and 0~16.7%, respectively (19). Vascular and intestinal damage were the most common intraoperative complications. In this study, three cases of vascular injury (3.85%) occurred in the TU-LESS group, while three cases of intestinal injury (3.85%) and one case of vascular injury (1.28%) occurred in the MLS group.

According to the literature, the incidence of incisional hernia in TU-LESS surgery is around 0~13.3%, while it is about 0~4.7% in multi-port laparoscopic surgery (24–27). Gunderson et al. (27) retrospectively analyzed 211 patients who underwent SPL for benign or malignant gynecological disorders and found that 2.4% of them developed an umbilical hernia 16 months later. Pollard et al. (28) showed that the incidence rate of postoperative umbilical incisional hernia with TU-LESS was 0.2%, compared to 1.6% for traditional porous laparoscopy, in research involving 3989 patients who underwent the procedure. Previous TU-LESS research has linked the development of postoperative umbilical hernia to a higher BMI (25 kg/m^2^) and previous history of hernia (29). Other risk factors include wound infection, ASA grading III or IV, diabetes, hypertension, longer operation time, and dilation of incision for specimen collection (7).

To close the umbilical incision, Zheng Ying et al. (18) advocated “Zheng’s anchor suturing approach” to limit the risk of slippage, this procedure uses continuous sutures to close the fascia layer, and the sutures at both ends of the closed layer are linked with the anchoring point suture. We followed up on 5523 patients who had TU-LESS surgery for various gynecologic illnesses at our facility and discovered that 6 of them (0.11%) had umbilical hernia 3 to 8 months after the operation. For all TU-LESS patients in this study, “Zheng’s anchoring suturing technique” was adopted for umbilical sutures. And there were no cases of umbilical hernia in any of our research groups.

Although SPL surgery has been evaluated in gynecologic oncology for several years, it has yet not been widely adopted by gynecologic oncologists. The main reason for the limited application of single-port laparoscopy in gynecologic oncology is the increased difficulty of surgery through a single surgical incision. SPL’s optical field was limited to a single aperture, the single umbilical channel, mutual instrument interference, and the lack of a surgical triangle make it difficult to apply for gynecological malignant tumors. To reach the stable stage of surgery, a surgeon needs a particular quantity of surgical experience and a cross-learning curve. Barnes et al. (11) reported a drastic improvement in surgical time could be seen after approximately the first 20 cases. In addition, several scholars noted a learning curve of 30 patients as the surgeon adapted to the technique (30). According to our experience, the collision of instruments could be avoided by using instruments of different lengths and articulating instruments during TU-LESS operation. The ultrasonic dissector should be kept well away from the blood artery wall when doing lymph node resection, and the risk of TU-LESS could be reduced by operating cautiously and avoiding violent maneuvers.

Recently, sentinel lymph node biopsy (SLNB) has been recommended by many guidelines for early-stage low-risk patients (6, 31). According to the 2018 NCCN recommendations, the SLNB might be used in high-risk EC patients. With the growing acceptance and increased adoption of sentinel lymph node biopsy by gynecologists, the scope of endometrial cancer staging will be reduced, and the learning curve via a single-port platform will be greatly shortened. TU-LESS surgery of endometrial cancer may be a viable option for both patients and surgeons, yielding favorable clinical benefit-risk evaluations. Our team pioneered the validation of sentinel lymph node biopsy at our center during the study period. In the TU-LESS cohort, 12 patients underwent sentinel lymph node biopsy, including 10 cases with the subsequent continuation of pelvic lymph node dissection and 1 case with pelvic plus para-aortic lymph node dissection, while the other had just sentinel lymph node biopsy. As it was the initial period, we completed lymph node dissection as scheduled for the patient after performing the sentinel lymph node biopsy.

Investigations of oncological outcomes are not often investigated. To the best of our knowledge, the largest sample size of 284 TU-LESS EC patients from multi-center had been compared with the other 866 patients (214 MLS, and 652 robot surgery). It was found that the surgical platform did not affect the patients’ progression-free survival or overall survival (4). The median follow-up time in the literatures was between 9 and 36 months (**Table 5**). The median follow-up time in our TU-LESS and MLS groups was 45 months and 43 months, respectively. Actually, we present a relatively longer median follow-up. There was no difference in overall survival and disease-free survival between patients who underwent multi-port laparoscopic surgery and those who underwent single-port laparoscopic surgery in our cohort. However, the follow-up period was not long enough to fully understand the mortality and recurrence rates for long-term survival.

**Table 5:** Literature review of LESS procedure in EC survival.

Authors	Year	Type of study	No. of cases	FIGO stage	Median lymph nodes (n)	Oncological outcomes			
						Recurrence	DOD	Median follow-up (month)	Ovrall survival
Corrado G (22)	2016	R	50	I-IIIA	14(5-20)	0	0	36 m (16-62)	NR
Barnes H (11)^ ^	2017	R	110	I-IV	30 PL(2-40), 15 PAO(1-29)	6 (5.4%)	2 (1.8%)	9.9m	
Chambers LM (4)	2019	R	284	IA-IVB	NR			31.1m (0.5-86.3)	5 years PFS: 85.2%, 5 years OS: 91.8%;
Our study	2023	R	78	I-IV	23 PL(16-28) 2 PAO (0-6)	5(6.41%)	1(1.28%)	45m (20-66)	

R, Retrospective; DOD, Dead of disease; NR, No reported; PL, Pelvic lymph nodes; PAO, Para-aortic lymph nodes.

The advantages of this study include this is an experienced tertiary gynecological tumor center, and all TU-LESS operations were provided by gynecological oncologists who are very experienced in laparoscopic gynecological tumor surgery. Selection bias is mitigated by the fact that the TU-LESS case series includes consecutive patients. Furthermore, to our knowledge, this is the biggest retrospective case-matched control study using TU-LESS surgery for endometrial cancer by a surgeon in one institution center, it is trustworthy proof that the procedure is safe and practicable in gynecologic oncology practice. This study has limitations that are also worth discussing. First, this is a retrospective study and the follow-up was not standard. Physical examinations, for example, could be performed by multiple medical facilities, resulting in an underestimation of postoperative problems and hernia incidence rates. As just one surgeon completed all of the TU-LESS operations, this presents another constraint; it is also possible that the results recorded do not apply to all surgeons. While it would be challenging to conduct randomized research, a prospective observational analysis of patients experiencing diverse minimally invasive modalities over multiple centers would be a helpful next way to ascertain whether one modality is preferable to another and to confirm whether the increased technical difficulties are merited for routine usage in clinical practice.

With the introduction of the da Vinci robotic system, the combination of a robot system and a single port platform will overcome many of the challenges in single-port surgery, such as instrument crowding and the need for highly advanced laparoscopic skills (32, 33). However, robotic surgery remains relatively expensive, single-port laparoscopic surgery may be a more relevant option for our patients, but it does pose a challenge to the surgeon’s surgical skills. Several studies have confirmed the safety and feasibility of vNOTES for staging surgery and sentinel lymph node biopsy in early endometrial cancer (34, 35), but large sample RCTs are needed to assess feasibility and safety, and most importantly, long-term survival outcome.

In conclusion, the current study reaffirms prior evidence that TU-LESS is a feasible and safe option for endometrial cancer surgery. With the popularity of sentinel lymph node biopsy in endometrial cancer staging surgery, TU-LESS endometrial cancer surgery may be an effective alternative for both patients and surgeons.

## Data availability statement

The original contributions presented in the study are included in the article/supplementary material. Further inquiries can be directed to the corresponding author.

## Ethics statement

The studies involving humans were approved by West China Second University Hospital’s Medical Ethics Committee. The studies were conducted in accordance with the local legislation and institutional requirements. Written informed consent for participation was not required from the participants or the participants’ legal guardians/next of kin in accordance with the national legislation and institutional requirements. Written informed consent was obtained from the individual(s) for the publication of any potentially identifiable images or data included in this article.

## Author contributions

YZ, YW and FY contributed to the conception of the study, and revision of the manuscript. XY performed the data collection and wrote the manuscript. YW, QW, LM, KW contributed to the analysis and performed the analysis with constructive discussions. NW contributed significantly to manuscript preparation. All authors contributed to the article and approved the submitted version.
